# Kinesiophobia phenotypes and fall history in older adults with osteoporosis: a cross-sectional study using latent profile analysis

**DOI:** 10.3389/fendo.2026.1904725

**Published:** 2026-08-21

**Authors:** MengPing Tian, Ling Li, WanQiu Lv, Gang Deng, Yang Ni, Jian Xiong

**Affiliations:** 1 School of Basic Medical Sciences & School of Nursing, Chengdu University, Chengdu, China; 2 Clinical Medical College & Affiliated Hospital of Chengdu University, Chengdu, China; 3 Mianyang People’s Hospital, Mianyang, China

**Keywords:** accidental falls, aging, fear of movement, fear-avoidance, latent profile analysis

## Abstract

**Objective:**

Falls are a leading cause of morbidity among older adults with osteoporosis. While traditional risk assessment focuses on biological factors such as bone mineral density (BMD), psychological factors—particularly kinesiophobia—have received increasing attention. However, most studies conceptualize kinesiophobia as a continuous construct, potentially overlooking clinically meaningful heterogeneity. This study aimed to identify kinesiophobia phenotypes and examine their association with fall history in older adults with osteoporosis.

**Methods:**

This cross-sectional study included 306 older adults with osteoporosis recruited from a tertiary hospital. Kinesiophobia was assessed using the Tampa Scale for Kinesiophobia (TSK-17) and the Kinesiophobia Causes Scale (KCS). Latent profile analysis (LPA) was performed to identify distinct kinesiophobia phenotypes based on standardized KCS scores. Multivariable logistic regression was used to evaluate the association between phenotypes and fall history, adjusting for age, body mass index (BMI), BMD, prognostic nutritional index (PNI), and the log-transformed systemic immune-inflammation index (log-SII). Model performance was assessed using the area under the receiver operating characteristic curve (AUC) with bootstrap validation.

**Results:**

Among 306 participants (mean age, 74.9 years; 77.1% women), 232 (75.8%) exhibited kinesiophobia and were included in the LPA. Three phenotypes were identified: sensitized, resilient, and frail. The frail phenotype was independently associated with a significantly higher odds of prevalent falls (OR, 2.93; 95% CI, 1.44–5.96; P = .003), whereas the sensitized phenotype was not. Notably, bone mineral density was not independently associated with fall history after adjustment (P = .735). The predictive model demonstrated moderate discrimination (AUC = 0.693; bootstrap AUC = 0.692), with improved performance after inclusion of kinesiophobia phenotypes.

**Conclusion:**

Kinesiophobia is a heterogeneous construct in older adults with osteoporosis. The frail kinesiophobia phenotype is associated with a substantially higher odds of prevalent falls, independent of traditional biological markers. These findings suggest that psychological and behavioral phenotyping may enhance fall history stratification and support more targeted prevention strategies in geriatric populations.

## 1 Introduction

Global population aging has substantially increased the burden of osteoporosis (1), which affects approximately 19.7% of the population worldwide and more than 35% of older women (2). In China, where population aging is accelerating rapidly, over 30% of adults aged ≥65 years are affected (3). Osteoporosis represents a systemic skeletal pathology marked by diminished bone density and deterioration of the bone’s microstructural integrity (4). Fragility fractures, particularly those occurring at the hip and spine, impose a substantial burden on older adults, manifesting as increased morbidity and mortality alongside heightened healthcare costs (5).

Falls represent the leading precipitating factor for osteoporotic fractures, with studies showing that 86–95% of low-trauma fractures in Western community-dwelling elderly are attributable to falls (6). In addition to traditional physiological factors such as impaired balance, muscle weakness, and comorbidities, psychological factors have increasingly been recognized as important contributors to fall history (7, 8). A key psychological contributor is kinesiophobia, a debilitating condition marked by an irrational and exaggerated fear of movement due to perceived susceptibility to pain or harm (9). Recent studies have reported that kinesiophobia affects 57.0% to 75.6% of older adults with osteoporosis (10, 11), a prevalence substantially higher than that observed in healthy populations, and is associated with poorer physical function and increased fear of falling (12, 13). Kinesiophobia may contribute to falls through fear-related activity avoidance and physical inactivity, which are associated with poorer muscle strength, balance, gait, and functional performance in older adults (14, 15). In individuals with osteoporosis, kinesiophobia is also associated with greater concern about falling and poorer physical function, supporting its potential relevance to fall vulnerability (16).

Although psychological factors have received increasing attention in musculoskeletal health, previous studies of kinesiophobia in older adults and individuals with osteoporosis have predominantly used variable-centered analyses, operationalizing kinesiophobia as a continuous total or domain score (11, 13, 17).However, fear-related responses to movement may vary substantially across individuals. Some patients may exhibit high fear but maintain functional capacity, whereas others may demonstrate low fear yet experience severe functional limitations (17, 18). This heterogeneity suggests that distinct psychological or behavioral phenotypes may exist within the broader construct of kinesiophobia (19). Latent profile analysis (LPA) and related person-centered approaches offer a robust framework for detecting empirically derived subgroups based on shared response profiles. The application of such techniques in behavioral medicine and gerontology has grown substantially, as they facilitate the discovery of clinically relevant phenotypes often masked by traditional analytic methods (20, 21).

Understanding heterogeneity in kinesiophobia may be particularly important in osteoporosis because psychological fear may interact with physical vulnerability, such as sarcopenia, impaired balance, and fracture risk (13). Existing evidence indicates, for example, that older adults with increased fracture risk and sarcopenia tend to exhibit greater fear of movement and elevated fall history (22). However, whether distinct kinesiophobia phenotypes exist among osteoporotic patients and how such phenotypes may relate to fall history remain largely unexplored.

In this context, the present study applied LPA to identify distinct kinesiophobia phenotypes among older adults with osteoporosis and to examine their association with fall history. By characterizing heterogeneity in fear-related behavioral patterns, this study aims to provide a more nuanced understanding of psychological vulnerability and to inform targeted strategies for fall prevention in this high-risk population.

## 2 Methods

### 2.1 Study design and participants

This cross-sectional investigation was conducted at the Department of Orthopedics, Affiliated Hospital of Chengdu University, between January and November 2025, with participants recruited via a convenience sampling strategy. Consecutive patients with a clinical diagnosis of osteoporosis were screened for eligibility during the study period. Inclusion criteria were:1) age ≥60 years;2) diagnosis of osteoporosis based on a trabecular bone mineral density (BMD) <80 mg/cm³ measured using quantitative computed tomography (QCT);3) ability to communicate and complete questionnaire assessments independently or with assistance;4) provision of written informed consent. Exclusion criteria included:1) severe cognitive impairment or diagnosed dementia;2) severe neurological or psychiatric disorders that could interfere with mobility or questionnaire completion;3) incomplete clinical or laboratory data. The protocol for this research underwent formal review and received ethical authorization from the Affiliated Hospital of Chengdu University’s Institutional Review Board (Reference: PJ2025-092-02). Each participant provided voluntary written consent prior to their enrollment in the study. Furthermore, all experimental methodologies were executed in strict accordance with the ethical standards established by the Declaration of Helsinki.

### 2.2 Sample size considerations

In accordance with the events-per-variable (EPV) principle for multivariable logistic regression, a conservative EPV of 10 was adopted to ensure model stability. With seven predictors planned (including three latent classes as dummy variables), a minimum of 70 fall events was required (23). Based on a previously reported annual fall incidence of 32.4%–40.5% in older adults with osteoporosis, the estimated sample size ranged from 173 to 217 participants (24). After accounting for a 5% rate of invalid responses, the final target sample size was determined to be 183 to 229 participants. A total of 306 participants were screened, of whom 232 met the criterion for clinically significant kinesiophobia, defined as a score of ≥37 on the 17-item Tampa Scale for Kinesiophobia (TSK-17), and were included in the LPA to explore heterogeneity among affected individuals. The remaining 74 participants without kinesiophobia were retained as an external reference group for comparative analyses.

### 2.3 Measurements

#### 2.3.1 Kinesiophobia

Kinesiophobia was assessed using the Chinese version of the TSK-17. The instrument consists of 17 items rated on a 4-point Likert scale, yielding a total score ranging from 17 to 68. Higher total scores indicate greater fear of movement (25) Consistent with previous research, clinically significant kinesiophobia was defined as a TSK-17 score of ≥37 (26).

The Chinese version of the Kinesiophobia Causes Scale (KCS) was used to assess the factors underlying fear of movement. The original scale was developed by Knapik et al. (27) and was subsequently cross-culturally adapted and validated in a Chinese population by Guo et al. (28). The KCS comprises 20 items rated on a 5-point Likert scale, ranging from 1 to 5. Items 1–11 assess biological causes of kinesiophobia, whereas items 12–20 assess psychological causes. The biological-domain score (KCS1) and psychological-domain score (KCS2) were calculated by averaging the corresponding item scores, with each domain score ranging from 1 to 5. Higher domain scores indicate a greater contribution of biological or psychological factors to fear of movement. The KCS1 and KCS2 domain scores were standardized as z scores before latent profile analysis.

#### 2.3.2 Pain assessment

Pain intensity was assessed using a 0–10 Visual Analog Scale (VAS). Participants indicated their perceived pain on a 10-cm horizontal line, anchored by 0 (“no pain”) and 10 (“worst imaginable pain”). This instrument has been previously validated by Price et al. as a reliable metric for chronic pain assessment (29).

#### 2.3.3 Functional status

Functional status was assessed using the Activities of Daily Living (ADL) scale (30), a widely used instrument for evaluating independence in daily activities among older adults. This scale has demonstrated excellent reliability and validity in geriatric and rehabilitation populations.

#### 2.3.4 Biological markers

Two systemic biomarkers were included to characterize physiological vulnerability.

Routine laboratory parameters, including serum albumin and peripheral blood lymphocyte count, were obtained from the medical records. The prognostic nutritional index (PNI), an indicator of nutritional and immune status, was calculated as serum albumin (g/L) + 5 × peripheral blood lymphocyte count (×10^9^/L) (31). The systemic immune-inflammation index (SII), a composite indicator of systemic inflammatory and immune status (32), was calculated as platelet count (×10^9^/L) × neutrophil count (×10^9^/L)/lymphocyte count (×10^9^/L) (33, 34). Because SII exhibited a right-skewed distribution, it was log-transformed before analysis and is hereafter referred to as log-SII.

#### 2.3.5 Clinical and demographic variables

Variables included age, sex, education level, marital status, body mass index (BMI), BMD, comorbidities, and fall history. Volumetric trabecular BMD (mg/cm³) was measured at the L1–L4 lumbar vertebrae using QCT. QCT was selected because volumetric BMD measurements are relatively less affected by degenerative changes commonly observed in older adults (35, 36).

#### 2.3.6 Fall assessment

Falls were defined according to established international criteria as an unexpected event in which the participant comes to rest on the ground, floor, or a lower level (e.g., stroke or seizures) or external force (37). Participants reported their fall history over the preceding 12 months through a binary “yes/no” query. Subsequently, the frequency and distribution of these events were categorized across the identified kinesiophobia phenotypes.

### 2.4 Statistical analysis

Statistical analyses were performed using R software version 4.3.2. Continuous variables were summarized as the mean ± standard deviation (SD), whereas categorical variables were presented as frequencies and percentages. Between-group differences were assessed using one-way analysis of variance or chi-square tests, with Fisher’s exact test using simulated P values applied to sparse categorical data. Effect sizes were quantified using eta-squared (η²) and bias-corrected Cramér’s V.

LPA was conducted among participants with a TSK-17 score of ≥37 and complete KCS1 and KCS2 data. Standardized KCS1 and KCS2 scores were used as continuous indicators. One- to three-profile models were estimated using the tidyLPA package with the mclust estimation engine, assuming equal indicator variances across profiles and zero covariance. No predefined KCS cut-off values were applied; participants were assigned to the profile with the highest posterior membership probability. Model selection was based on the AIC, BIC, SABIC, entropy, profile size, and clinical interpretability. The retained profiles were ordered by their mean raw KCS1 scores and labeled sensitized, resilient, and frail.

Multivariable binary logistic regression was used to examine the association between kinesiophobia phenotypes and fall history during the preceding 12 months. Candidate covariates were initially examined in preliminary univariable analyses and were further considered according to clinical relevance and model parsimony. Age, BMI, BMD, PNI, log-transformed SII, and phenotype were entered simultaneously, with the resilient phenotype as the reference category. No automated forward, backward, or stepwise selection procedure was used. Fall history during the preceding 12 months constituted the dependent variable and was therefore not included as an additional covariate; information on falls preceding this period was unavailable. Results were reported as ORs with 95% CIs.

## 3 Results

### 3.1 Participant characteristics

**Figure 1:**
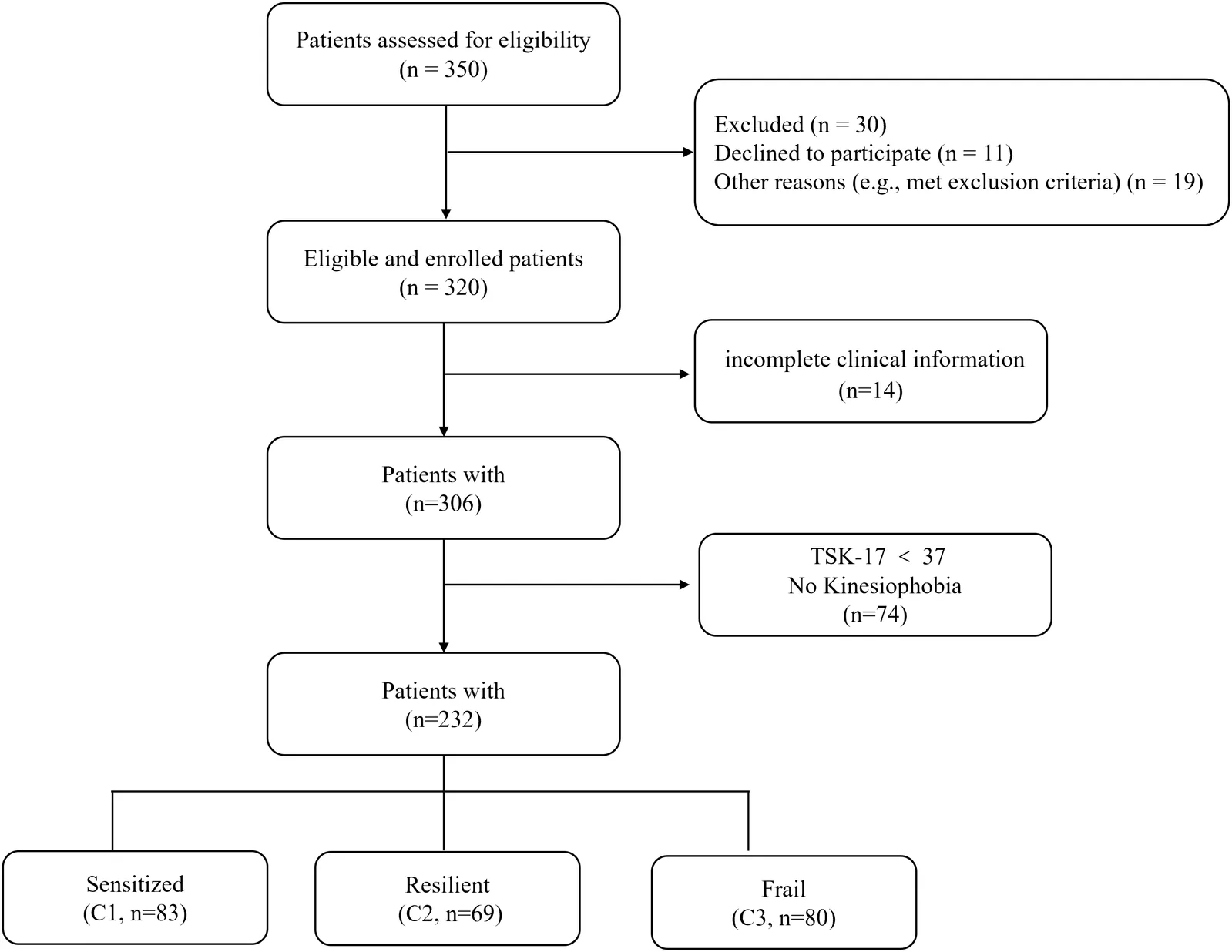
Flowchart of participant selection.

A total of 306 participants were included, of whom 232 (75.8%) met the criterion for clinically significant kinesiophobia and were included in the LPA; the remaining 74 participants (24.2%) were classified as having no clinically significant kinesiophobia and served as the reference group (**Figure 1**). Baseline characteristics across kinesiophobia phenotypes are presented in **Table 1**. The mean (SD) age was 74.94 (9.62) years, and 236 participants (77.1%) were women. Significant between-group differences were observed for age (P = .025), marital status (P = .002), bone mineral density (BMD) (P = .001), prognostic nutritional index (PNI) (P = .010), and activities of daily living (ADL) (P = .016). The frail phenotype was characterized by older age, lower BMD, poorer nutritional status, and reduced functional capacity. However, effect sizes were small (η² range, 0.031–0.051), indicating modest between-group differences. Regarding fall history, 172 participants (56.2%) reported at least one fall in the past 12 months. The prevalence of falls varied significantly across groups (P = 0.004), with the highest rate observed in the frail phenotype (57/80, 71.3%), followed by the sensitized phenotype (46/83, 55.4%), the non-kinesiophobia group (40/74, 54.1%), and the lowest rate in the resilient phenotype (29/69, 42.0%).

**Table 1:** Baseline characteristics of participants according to kinesiophobia phenotypes.

Characteristics	Category	Overall (n=306)	Non-kinesiophobia (n=74)	Kinesiophobia phenotypes			P value	Effect size
				Sensitized (C1, n=83)	Resilient (C2, n=69)	Frail (C3, n=80)		
Demographic characteristics								
Age, years		74.94 (9.62)	73.14 (8.21)	75.40 (9.72)	73.52 (9.62)	77.35 (10.31)	0.025	0.031 (η²)
Sex	Men	70 (22.9)	22 (29.7)	16 (19.3)	18 (26.1)	14 (17.5)	0.231	0.065 (V)
	Women	236 (77.1)	52 (70.3)	67 (80.7)	51 (73.9)	66 (82.5)		
Education	Primary or below	162 (52.9)	42 (56.8)	45 (54.2)	29 (42.0)	46 (57.5)	0.499	0.000 (V)
	Secondary	141 (46.1)	31 (41.9)	37 (44.6)	39 (56.5)	34 (42.5)		
	Tertiary and above	3 (1.0)	1 (1.4)	1 (1.2)	1 (1.4)	0 (0.0)		
Marital status	Married	233 (76.1)	60 (81.1)	60 (72.3)	58 (84.1)	55 (68.8)	0.002	0.158 (V)
	Widowed	61 (19.9)	8 (10.8)	23 (27.7)	7 (10.1)	23 (28.7)		
	Unmarried	12 (3.9)	6 (8.1)	0 (0.0)	4 (5.8)	2 (2.5)		
Body composition and bone status								
BMI, kg/m²		23.69 (3.97)	23.77 (3.11)	23.99 (4.95)	23.66 (3.34)	23.33 (4.09)	0.767	0.004 (η²)
BMD, mg/cm³		51.86 (17.97)	58.39 (15.53)	51.28 (19.46)	51.06 (17.72)	47.13 (17.25)	0.001	0.051(η²)
Clinical characteristics								
Comorbidities	0-1	115 (37.6)	30 (40.6)	25 (30.1)	32 (46.4)	28 (35)	0.250	0.051 (V)
	≥2	191 (62.4)	44 (59.4)	58(69.9)	37 (53.6)	52(65)		
Nutritional and inflammatory markers								
Serum albumin, g/L		39.52 (4.89)	39.59 (4.41)	39.79 (4.46)	40.50 (4.59)	38.24 (5.75)	0.035	0.028 (η²)
Lymphocyte count, ×10^9^/L		1.28 (0.56)	1.32 (0.52)	1.27 (0.57)	1.33 (0.59)	1.20 (0.57)	0.432	0.009 (η²)
Platelet count, ×10^9^/L		197.37 (64.55)	203.62 (65.30)	191.78 (56.97)	196.00 (67.32)	198.46 (69.01)	0.719	0.004 (η²)
Neutrophil count, ×10^9^/L		5.67 (5.45)	5.51 (2.94)	5.54 (3.09)	5.17 (2.62)	6.45 (9.61)	0.507	0.008 (η²)
PNI		45.92 (6.10)	46.20 (5.22)	46.17 (5.72)	47.39 (6.06)	44.12 (6.89)	0.010	0.037 (η²)
log-SII		6.65 (0.89)	6.63 (0.79)	6.66 (0.82)	6.49 (0.88)	6.78 (1.04)	0.263	0.013 (η²)
Functional, pain, and fall characteristics								
ADL score		63.66 (25.22)	65.14 (23.52)	61.87 (26.08)	70.80 (24.07)	58.00 (25.64)	0.016	0.034 (η²)
VAS score		2.33 (1.60)	2.24 (1.61)	2.59 (1.75)	1.94 (1.43)	2.46 (1.52)	0.071	0.023 (η²)
Falls (past 12 months)	Yes	172 (56.2%)	40 (54.1%)	46 (55.4%)	29 (42.0%)	57 (71.3%)	0.004	0.182 (V)
Kinesiophobia-related characteristics								
KCS biological-domain score		2.79 (0.92)	2.98 (0.93)	3.74 (0.44)	2.67 (0.30)	1.74 (0.33)	<0.001	
KCS psychological-domain score		2.83 (0.92)	2.92 (0.85)	3.74 (0.81)	2.69 (0.31)	1.90 (0.30)	<0.001	

Continuous variables are presented as mean (standard deviation), and categorical variables are presented as n (%). P values were calculated using one-way analysis of variance for continuous variables and chi-square tests for categorical variables.

ADL, Activities of Daily Living; BMD, bone mineral density; BMI, body mass index; KCS, Kinesiophobia Causes Scale; PNI, prognostic nutritional index; SII, systemic immune-inflammation index; VAS, Visual Analog Scale.

Effect sizes: η² denotes eta-squared for continuous variables, and V denotes Cramér’s V for categorical variables.

KCS scores are presented as raw domain means (range: 1–5).

### 3.2 Identification of kinesiophobia phenotypes

**Figure 2:**
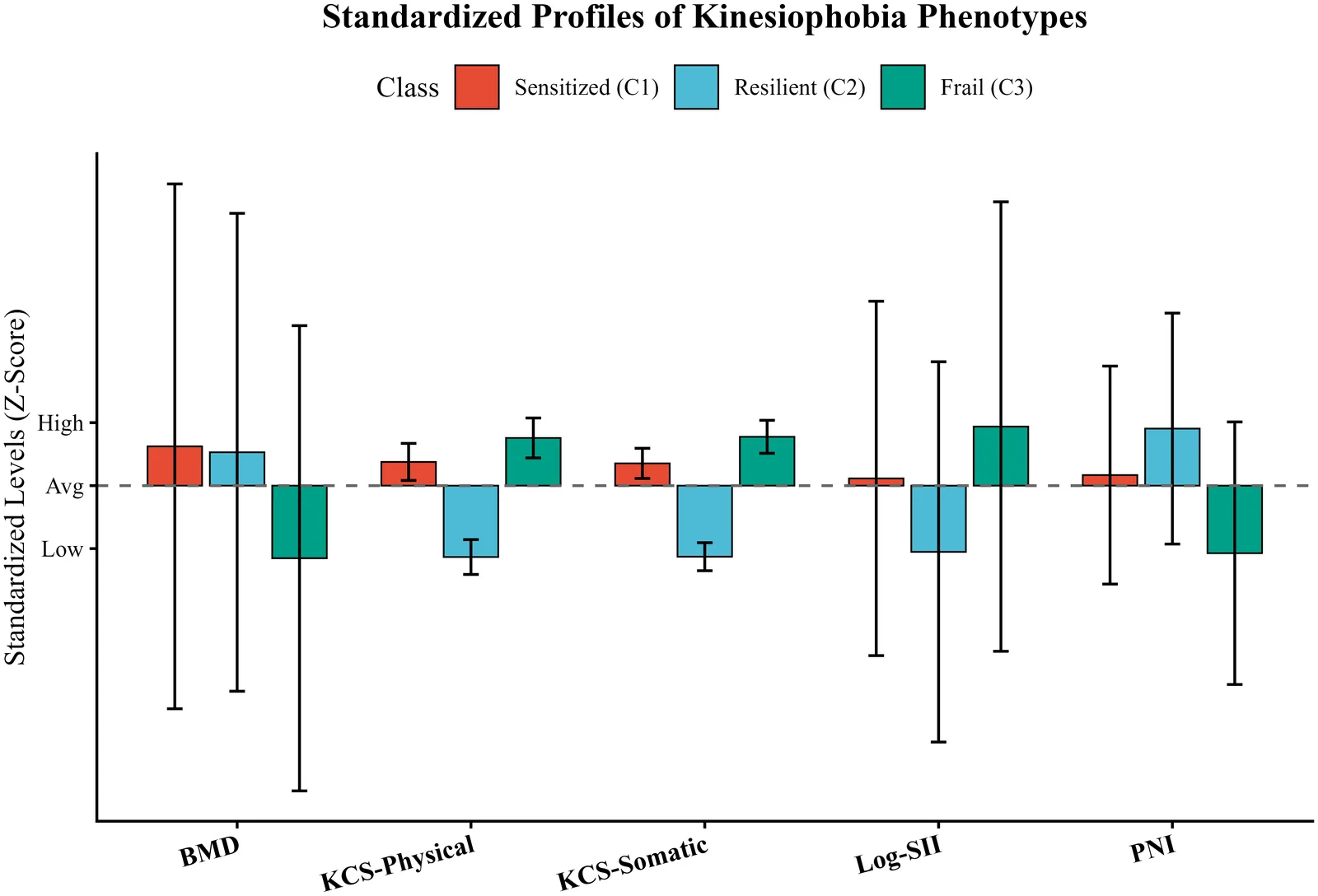
Standardized profiles of kinesiophobia phenotypes. Standardized mean levels of BMD, KCS biological-domain score, KCS psychological-domain score, log-SII, and PNI across the sensitized, resilient, and frail phenotypes. Values are presented as z scores, with zero representing the overall sample mean.

Latent profile analysis models with one to three classes were estimated (**Table 2**). Model fit indices improved with increasing class numbers, as reflected by decreasing AIC, BIC, and SABIC values, and acceptable entropy (**Figure 2**). The three-class solution was ultimately selected, as it demonstrated the optimal balance between statistical fit criteria, clinical relevance, and cluster-specific sample sizes. This determination was grounded in a holistic assessment of both model performance and interpretability. The identified phenotypes were labeled as: sensitized (Class 1), resilient (Class 2) and frail (Class 3). The sensitized phenotype was the largest group (n = 83, 35.8% of the 232 participants with kinesiophobia), followed by the frail (n = 80, 34.5%) and resilient (n = 69, 29.7%) phenotypes.

**Table 2:** Model fit indices for latent profile analysis.

Number of classes	AIC	BIC	SABIC	Entropy
1	1,322.77	1,336.56	1,323.88	1.000
2	1,139.91	1,164.04	1,141.85	0.849
3	1,104.97	1,139.44	1,107.74	0.787

Lower values of AIC, BIC, and SABIC indicate better model fit. Entropy values closer to 1.0 indicate clearer class separation. The bootstrap likelihood ratio test (BLRT) compares model fit between k and k–1 class solution.

AIC, Akaike information criterion; BIC, Bayesian information criterion; SABIC, sample-size adjusted Bayesian information criterion; BLRT, bootstrap likelihood ratio test.

### 3.3 Phenotype characteristics

Phenotype-specific KCS domain scores and clinical characteristics are summarized in **Table 1**. As shown in **Table 1**, the sensitized phenotype exhibited the highest biological-domain and psychological-domain KCS scores, with mean scores of 3.74 and 3.74, respectively. In contrast, the frail phenotype showed the lowest KCS domain scores but the least favorable clinical profile, including the lowest BMD, serum albumin, PNI, and ADL scores and the highest prevalence of falls. The resilient phenotype demonstrated the most favorable nutritional and functional profile, including the highest PNI and ADL scores. These patterns suggest that the identified phenotypes reflect multidimensional differences in psychological, nutritional, and functional status rather than the intensity of fear of movement alone.

### 3.4 Association between phenotypes and fall history

**Figure 3:**
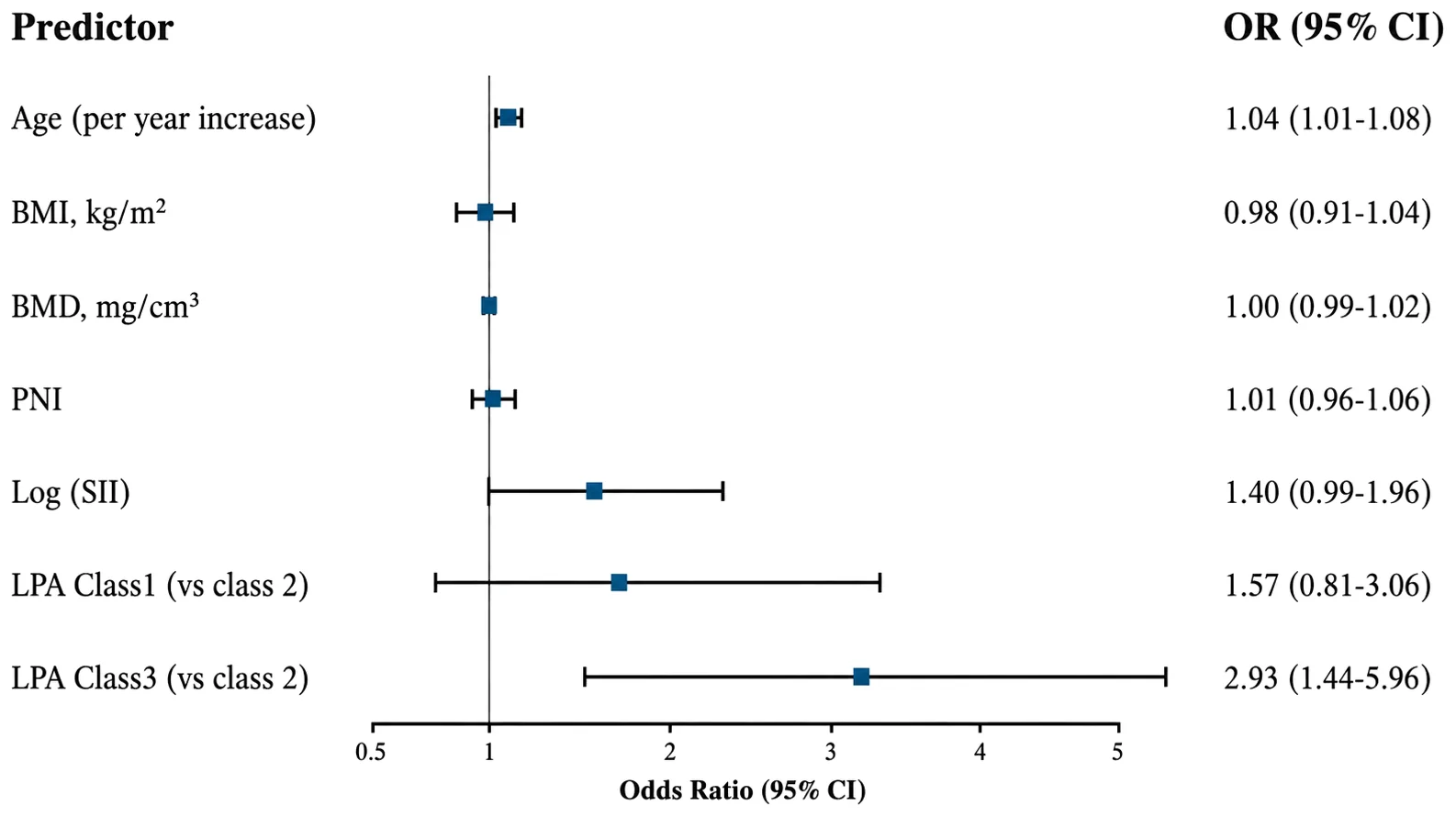
Forest plot of multivariable logistic regression. Forest plot of multivariable logistic regression for fall history. Adjusted odds ratios (ORs) with 95% confidence intervals (CIs) are shown for each predictor. The resilient phenotype (LPA Class 2) served as the reference. The vertical dashed line indicates OR = 1.0.

Multivariable logistic regression results are presented in **Table 3** and **Figure 3**. After adjustment, older age was associated with higher odds of reporting at least one fall during the preceding 12 months (OR, 1.04; 95% CI, 1.01–1.08; P = .021). Compared with the resilient phenotype, the frail phenotype was associated with higher odds of fall history (OR, 2.93; 95% CI, 1.44–5.96; P = .003), whereas the sensitized phenotype was not significantly associated with fall history. BMD was not independently associated with fall history after adjustment (P = .735).

**Table 3:** Multivariable logistic regression analysis of factors associated with fall history.

Variable	Beta	OR (95CI)	P
Age (per year increase)	0.040	1.04 (1.01-1.08)	0.021
BMI, kg/m²	-0.024	0.98 (0.91-1.04)	0.481
BMD, mg/cm³	0.003	1.00 (0.99-1.02)	0.735
PNI	0.005	1.01 (0.96-1.06)	0.834
Log (SII)	0.335	1.40 (0.99-1.96)	0.054
LPA Class1(VS class 2)	0.451	1.57 (0.81-3.06)	0.184
LPA Class3(VS class 2)	1.076	2.93 (1.44-5.96)	0.003

Reference group: Resilient phenotype (LPA class 2).

Odds ratios were obtained from a multivariable logistic regression model adjusting for demographic and clinical variables.

BMI, body mass index; BMD, Bone mineral density; PNI, prognostic nutritional index; SII, systemic immune-inflammation index; LPA, latent profile analysis.

### 3.5 Model performance and validation

**Figure 4:**
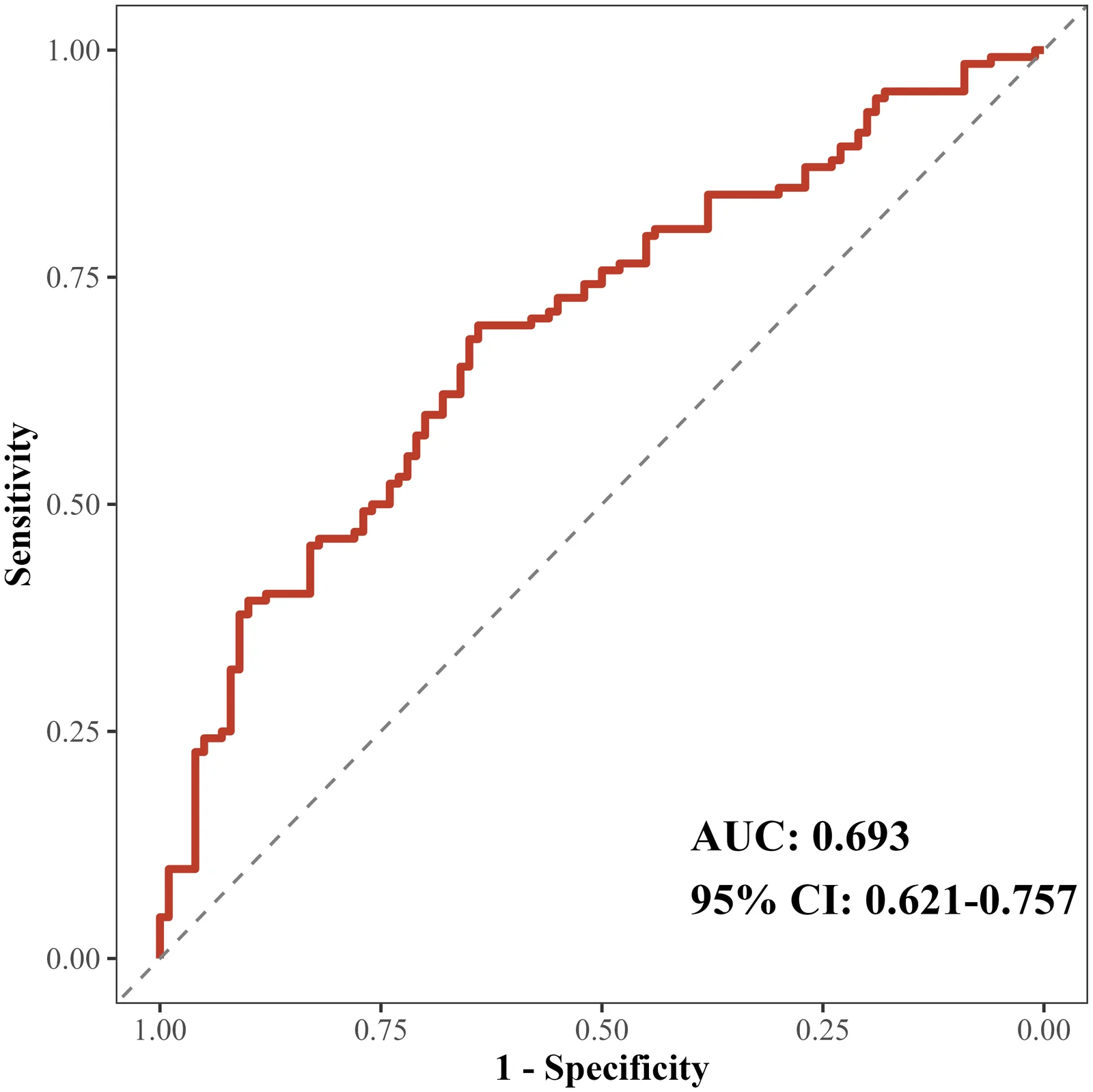
Receiver operating characteristic (ROC). Receiver operating characteristic (ROC) curve for the predictive model of fall history. The model included age, BMI, BMD, PNI, log-SII, and kinesiophobia phenotypes. AUC = 0.693 (95% CI: 0.621–0.757). Bootstrap validation (1,000 iterations) yielded AUC = 0.692.

Model performance is summarized in **Table 4** and **Figure 4**. The model demonstrated moderate discrimination (AUC = 0.693; bootstrap AUC = 0.692, 95% CI 0.621–0.757). Calibration was acceptable (Hosmer–LemeShow P = .226). No significant multicollinearity was observed (maximum VIF = 2.00). To further assess incremental predictive value, three models were compared: Model 1: demographic variables, Model 2: + biological markers and Model 3: + kinesiophobia phenotypes. Model 3 showed improved discrimination, highlighting the added predictive value of psychological phenotypes. Although the model demonstrated modest discrimination, it may provide complementary information regarding fall-related vulnerability. Further validation and incorporation of additional established fall-related factors are required before clinical application.

**Table 4:** Predictive performance and internal validation of the logistic regression model.

Performance metric	Value
AUC (Model C)	0.693
Nagelkerke R^2^	0.145
Hosmer-Lemeshow P	0.226
Maximum VIF	2.000
Bootstrap AUC (95% CI)	0.692 (0.621 - 0.757)

Model adjusted for age, BMI, BMD, PNI, log-SII, and LPA Class. Bootstrap based on 1000 resamples. Model discrimination was assessed using the area under the receiver operating characteristic curve (AUC). Calibration was evaluated using the Hosmer–Lemeshow goodness-of-fit test. Internal validation was performed using bootstrap resampling.

AUC, area under the receiver operating characteristic curve; VIF, variance inflation factor.

## 4 Discuss

Three distinct kinesiophobia phenotypes were identified in this cross-sectional study, and the frail phenotype was associated with nearly threefold higher odds of prevalent falls (OR, 2.93). Given the cross-sectional design, all reported associations should be interpreted as correlational rather than causal, and the proposed mechanistic pathways remain speculative. This magnitude of association is comparable to, or exceeds, several established fall history factors reported in prior meta-analyses, where effect sizes for individual predictors such as muscle weakness, balance impairment, and prior falls typically range from 1.5 to 2.5 (38–40). Notably, in the present study, this psychological phenotype remained a significant predictor after adjustment, whereas traditional biological markers such as BMD did not, suggesting that behavioral and psychological vulnerability may represent a more proximal determinant of fall history in this population. These findings underscore the potential importance of psychological phenotyping as a complementary dimension to traditional biological risk assessment.

The observed relationship between the frail kinesiophobia phenotype and heightened fall history may be attributable to multiple underlying mechanisms. First, excessive fear of movement may promote activity avoidance, leading to reduced muscle strength, impaired balance, and diminished physical performance. This pathway is consistent with the fear-avoidance model, which describes how persistent movement-related fear can lead to physical deconditioning and functional decline (41). Second, fear of falling may alter gait and postural control strategies. Experimental and observational studies have shown that individuals with elevated fear adopt more rigid or cautious movement patterns, which may paradoxically impair dynamic stability and increase the likelihood of falls (42, 43). Third, kinesiophobia may interact with broader geriatric syndromes such as frailty and sarcopenia. Recent cohort studies have reported that frailty is associated with a 2-to-3-fold increase in fall history, a magnitude strikingly similar to the effect observed for the frail kinesiophobia phenotype in this study (44–46), supporting the interpretation that these constructs may share overlapping pathways. This may also explain the counterintuitive finding that the frail phenotype demonstrated lower kinesiophobia scores yet higher fall history.

The present findings extend previous research on kinesiophobia in osteoporosis in several important ways. Previous research has indicated that kinesiophobia affects approximately 50% to 60% of individuals in this population, contributing to decreased engagement in physical activity and diminished quality of life (10, 12, 16). However, these studies have generally treated kinesiophobia as a continuous variable and have not quantified its impact on hard outcomes such as falls. By contrast, the present study demonstrates that when modeled as a phenotype, kinesiophobia is associated with nearly a threefold increase in fall history, highlighting its potential clinical significance. Moreover, the use of latent profile analysis is supported by recent studies in geriatric populations showing that phenotype-based approaches can identify subgroups with substantially different risks of adverse outcomes, often with effect sizes exceeding those observed in traditional analyses (47–49).

The observed association between the frail phenotype and fall history is also consistent with broader epidemiological evidence indicating that falls arise from the interaction of psychological, functional, and physiological factors. Meta-analyses have shown that fear of falling alone is associated with approximately a 1.8- to 2.2-fold increased risk of future falls (50, 51). The stronger association observed in the present study (OR ≈ 2.9) suggests that kinesiophobia phenotypes may capture a more severe or multidimensional behavioral vulnerability, incorporating both fear and its functional consequences. This interpretation is further supported by studies demonstrating that activity restriction and reduced movement variability are independently associated with increased fall history (52, 53).

Although BMD is a well-established predictor of fracture risk, its association with falls is relatively weak. Large epidemiological studies have consistently demonstrated that BMD explains only a small proportion of fall variance, with most predictive models achieving modest discrimination (AUC typically 0.60–0.70) (54, 55). In the present study, the overall model demonstrated similar performance (AUC, 0.693), yet the inclusion of kinesiophobia phenotypes contributed meaningful predictive value, whereas BMD was not independently associated with falls. These outcomes suggest that incorporating psychological and behavioral dimensions may improve risk stratification beyond traditional structural measures.

These findings have practical implications for clinical risk assessment and intervention design in geriatric care. First, the identification of kinesiophobia phenotypes may enable more precise risk stratification, particularly given that the magnitude of risk associated with the frail phenotype is comparable to established clinical predictors. Second, screening for kinesiophobia may provide incremental prognostic value beyond traditional assessments focused on physical performance. Third, intervention studies have demonstrated that exercise-based and cognitive-behavioral interventions can reduce fear of falling by 20%–30% and improve functional outcomes (56, 57), suggesting that targeting fear-avoidance behaviors may be an effective strategy for reducing fall risk. These findings challenge the conventional emphasis on structural bone measures and suggest that behavioral phenotyping may provide more proximal insights into fall history in older adults with osteoporosis.

## 5 Limitations

This study has several limitations. First, the single-center design and convenience sampling may limit the generalizability of the findings. Second, the cross-sectional design precludes determination of temporal direction or causality. Third, fall history during the preceding 12 months was self-reported and may have been affected by recall bias. Fourth, women accounted for 77.1% of the sample, which may limit generalizability to men. Fifth, medication burden, sarcopenia, objective balance impairment, physical activity levels, and fall history preceding the assessed 12-month period were not collected. In addition, candidate covariates were partly selected on the basis of univariable associations; therefore, residual confounding by measured or unmeasured fall-related factors cannot be excluded. Finally, the model showed only modest discrimination and limited explained variation and should not be regarded as a clinically validated prediction tool. Prospective multicenter studies incorporating comprehensive fall-related assessments are needed.

## 6 Conclusion

In this study, kinesiophobia was identified as a heterogeneous construct in older adults with osteoporosis, with three distinct phenotypes characterized using latent profile analysis. The frail phenotype was independently associated with a substantially higher odds of prevalent falls, whereas traditional biological markers such as bone mineral density were not significant predictors after adjustment. These findings suggest that psychological and behavioral vulnerability may play a more proximal role in fall history than structural bone measures. Incorporating kinesiophobia phenotyping into clinical assessment may improve risk stratification and support more targeted fall prevention strategies in this population. Future longitudinal studies are needed to confirm these associations and to evaluate the impact of phenotype-guided interventions.

## Data Availability

The original contributions presented in the study are included in the article/supplementary material. Further inquiries can be directed to the corresponding author.

## References

[1] Gianfredi V Nucci D Pennisi F Maggi S Veronese N and Soysal P . Aging, longevity, and healthy aging: the public health approach. Aging Clin Exp Res. (2025) 37:125. doi: 10.1007/s40520-025-03021-8 40244306 PMC12006278

[2] Xiao PL Cui AY Hsu CJ Peng R Jiang N Xu XH . Global, regional prevalence, and risk factors of osteoporosis according to the World Health Organization diagnostic criteria: a systematic review and meta-analysis. Osteoporos Int. (2022) 33:2137–53. doi: 10.1007/s00198-022-06454-3 35687123

[3] Wang L Yu W Yin X Cui L Tang S Jiang N . Prevalence of osteoporosis and fracture in China: the China Osteoporosis Prevalence Study. JAMA Netw Open. (2021) 4:e2121106. doi: 10.1001/jamanetworkopen.2021.21106 34398202 PMC8369359

[4] Walker MD Shane E . Postmenopausal osteoporosis. N Engl J Med. (2023) 389:1979–91. doi: 10.1056/NEJMcp2307353 37991856

[5] Zhu Q Ran C . Health outcomes and care needs after osteoporotic fractures in rural Chinese older adults: policy implications. Front Public Health. (2025) 13:1601892. doi: 10.3389/fpubh.2025.1601892 40636878 PMC12237659

[6] Rommers E De Pauw R Petrovic M and Cambier D . Epidemiology of falls in community-dwelling older adults in Europe: a systematic review and meta-analysis. Age Ageing. (2025) 54:afaf157. doi: 10.1093/ageing/afaf157 40479612

[7] Ellmers TJ Ventre JP Freiberger E Hauer K Hogan DB Lim ML . Does concern about falling predict future falls in older adults? A systematic review and meta-analysis. Age Ageing. (2025) 54:afaf089. doi: 10.1093/ageing/afaf089 40197783 PMC11976718

[8] Chen WC Li YT Tung TH Chen C and Tsai CY . The relationship between falling and fear of falling among community-dwelling elderly. Med (Baltimore). (2021) 100:e26492. doi: 10.1097/md.0000000000026492 34190176 PMC8257838

[9] Luque-Suarez A Martinez-Calderon J Falla D . Role of kinesiophobia on pain, disability and quality of life in people suffering from chronic musculoskeletal pain: a systematic review. Br J Sports Med. (2019) 53:554–9. doi: 10.1136/bjsports-2017-098673 29666064

[10] Lyu FF Ying H Zhang M Xia LR Liu Q and Cai L . Prevalence and influencing factors of kinesiophobia in older patients with primary osteoporosis: a cross-sectional survey. Geriatr Nurs. (2024) 57:58–65. doi: 10.1016/j.gerinurse.2024.03.007 38537554

[11] Tian M Lv W Miao X Chen C Xiong J and Zhang J . Association between kinesiophobia and bone mineral density in hospitalized older adults with osteoporosis: a cross-sectional study. Front Endocrinol (Lausanne). (2026) 17:1731122. doi: 10.3389/fendo.2026.1731122 41710406 PMC12909168

[12] Ahısha B . Kinesiophobia and associated factors in postmenopausal osteoporosis: a controlled study. Rev Assoc Med Bras (1992). (2024) 70:e20241042. doi: 10.1590/1806-9282.20241042 39630733 PMC11639509

[13] Alshahrani MS Reddy RS . Kinesiophobia, limits of stability, and functional balance assessment in geriatric patients with chronic low back pain and osteoporosis: a comprehensive study. Front Neurol. (2024) 15:1354444. doi: 10.3389/fneur.2024.1354444 38414551 PMC10897043

[14] Alpalhão V Cordeiro N Pezarat-Correia P . Kinesiophobia and fear avoidance in older adults: a systematic review on constructs and related measures. J Geriatr Phys Ther. (2022) 45:207–14. doi: 10.1519/jpt.0000000000000354 35939664

[15] Roberts HJ Johnson KM Sullivan JE and Hoppes CW . Fear of falling avoidance behaviour, but not fall history, is associated with balance and dynamic gait performance in community-dwelling older adults: a cross-sectional study. Physiother Can. (2025) 77:329–36. doi: 10.3138/ptc-2023-0043 42079693 PMC13134864

[16] Stubbs B Patchay S Soundy A and Schofield P . The avoidance of activities due to fear of falling contributes to sedentary behavior among community-dwelling older adults with chronic musculoskeletal pain: a multisite observational study. Pain Med. (2014) 15:1861–71. doi: 10.1111/pme.12570 25224385

[17] Alpalhão V Cordeiro N Pezarat-Correia P . Kinesiophobia and fear avoidance in older adults: a scoping review on the state of research activity. J Aging Phys Act. (2022) 30:1075–84. doi: 10.1123/japa.2021-0409 35303715

[18] Zhu S Xu Y Wang L Chen J and Luo A . Efficacy of cognitive behavioral therapy for kinesiophobia: a systematic review and meta-analysis of randomized controlled trials. J Pain Res. (2025) 18:3919–32. doi: 10.2147/jpr.S526179 40791870 PMC12336382

[19] Xinxia W Dalin K Juan J Mingzhe D Lixia Z Weiling F . Latent profile analysis and associated factors of kinesophobia in patients with hip fractures. BMC Psychol. (2026) 14(1):365. doi: 10.1186/s40359-026-04131-2 41680876 PMC12998125

[20] Gholi Zadeh Kharrat F Amar Kumar P Mehling W Strigo I Lotz J and Peterson TA . Biobehavioral phenotypes of chronic low back pain: psychosocial subgroup identification using latent profile analysis. Pain Med. (2026) 27:15–25. doi: 10.1093/pm/pnaf095 40711854 PMC12419233

[21] Lentz TA George SZ Manickas-Hill O Malay MR O'Donnell J Jayakumar P . What general and pain-associated psychological distress phenotypes exist among patients with hip and knee osteoarthritis? Clin Orthop Relat Res. (2020) 478:2768–83. doi: 10.1097/corr.0000000000001520 33044310 PMC7899410

[22] Saedon NI Lok JQ . Osteoporosis management and fall-related complications in older adults: a retrospective study from the UMMC falls clinic. BMC Geriatr. (2025) 25:1043. doi: 10.1186/s12877-025-06746-3 41275075 PMC12751816

[23] Peduzzi P Concato J Kemper E Holford TR and Feinstein AR . A simulation study of the number of events per variable in logistic regression analysis. J Clin Epidemiol. (1996) 49:1373–9. doi: 10.1016/s0895-4356(96)00236-3 8970487

[24] Yosef T Pasco JA Tembo MC Williams LJ and Holloway-Kew KL . Falls and fall-related injuries: prevalence, characteristics, and treatment among participants of the Geelong Osteoporosis Study. Front Public Health. (2024) 12:1454117. doi: 10.3389/fpubh.2024.1454117 39494080 PMC11527698

[25] Roelofs J van Breukelen G Sluiter J Frings-Dresen MHW Goossens M Thibault P . Norming of the Tampa Scale for Kinesiophobia across pain diagnoses and various countries. Pain. (2011) 152:1090–5. doi: 10.1016/j.pain.2011.01.028 21444153

[26] Wei X Xu X Zhao Y Hu W Bai Y and Li M . The Chinese version of the Tampa Scale for Kinesiophobia was cross-culturally adapted and validated in patients with low back pain. J Clin Epidemiol. (2015) 68:1205–12. doi: 10.1016/j.jclinepi.2015.07.003 26169840

[27] Knapik A Saulicz E Gnat R . Kinesiophobia - introducing a new diagnostic tool. J Hum Kinet. (2011) 28:25–31. doi: 10.2478/v10078-011-0019-8 23487514 PMC3592098

[28] Guo Y Liu Y Zheng Y Cai L Ren M and Zhai Y . Cross-cultural adaptation and validation of the kinesiophobia causes scale: a descriptive survey have undergone total knee arthroplasty in China. J Orthop Sci. (2025) 30:614–9. doi: 10.1016/j.jos.2024.09.006 39406564

[29] Price DD McGrath PA Rafii A and Buckingham B . The validation of visual analogue scales as ratio scale measures for chronic and experimental pain. Pain. (1983) 17:45–56. doi: 10.1016/0304-3959(83)90126-4 6226917

[30] Katz S Ford AB Moskowitz RW Jackson BA and Jaffe MW . Studies of illness in the aged. The index of ADL: a standardized measure of biological and psychosocial function. Jama. (1963) 185:914–9. doi: 10.1001/jama.1963.03060120024016 14044222

[31] Guo H Yang L Liu J Yu X Chen L and Huang Y . Prognostic nutritional index and the risk of postoperative complications after spine surgery: a meta-analysis. World Neurosurg. (2024) 185:e572–81. doi: 10.1016/j.wneu.2024.02.077 38382761

[32] Hu B Yang XR Xu Y Sun YF Sun C Guo W . Systemic immune-inflammation index predicts prognosis of patients after curative resection for hepatocellular carcinoma. Clin Cancer Res. (2014) 20:6212–22. doi: 10.1158/1078-0432.Ccr-14-0442 25271081

[33] Xu Y He X Zhang X Meng S Wang C Yuan Y . The predictive value of immune inflammation indexes for the risk of fracture in patients with osteoporosis: a systematic review and meta-analysis. Front Endocrinol (Lausanne). (2025) 16:1650895. doi: 10.3389/fendo.2025.1650895 41255530 PMC12620189

[34] Tu X Lin T Huang L Jiang T Zheng Q and Yue J . Diagnostic performance of the systemic immune-inflammation index (SII) and sarcopenia index (SI) in sarcopenia and their prognostic value for clinical outcomes in hospitalized older patients. BMC Geriatr. (2025) 25:691. doi: 10.1186/s12877-025-06346-1 40993523 PMC12462163

[35] Lin W He C Xie F Chen T Zheng G Yin H . Quantitative CT screening improved lumbar BMD evaluation in older patients compared to dual-energy X-ray absorptiometry. BMC Geriatr. (2023) 23:231. doi: 10.1186/s12877-023-03963-6 37069511 PMC10108496

[36] Zou D He X Shang Z Jin D and Li W . Osteoporosis screening using QCT-based cutoff value of Hounsfield units in patients with degenerative lumbar diseases. Eur Spine J. (2024) 33:4499–503. doi: 10.1007/s00586-024-08491-4 39297897

[37] The prevention of falls in later life. A report of the Kellogg International Work Group on the Prevention of Falls by the Elderly. Dan Med Bull. (1987) 34:1–24. 3595217

[38] Shao L Shi Y Xie XY Wang Z Wang ZA and Zhang JE . Incidence and risk factors of falls among older people in nursing homes: systematic review and meta-analysis. J Am Med Dir Assoc. (2023) 24:1708–17. doi: 10.1016/j.jamda.2023.06.002 37433427

[39] Jehu DA Davis JC Falck RS Bennett KJ Tai D Souza MF . Risk factors for recurrent falls in older adults: a systematic review with meta-analysis. Maturitas. (2021) 144:23–8. doi: 10.1016/j.maturitas.2020.10.021 33358204

[40] Montero-Odasso M van der Velde N Martin FC Petrovic M Tan MP Ryg J . World guidelines for falls prevention and management for older adults: a global initiative. Age Ageing. (2022) 51:afac205. doi: 10.1093/ageing/afac205 36178003 PMC9523684

[41] Vlaeyen JWS Linton SJ . Fear-avoidance and its consequences in chronic musculoskeletal pain: a state of the art. Pain. (2000) 85:317–32. doi: 10.1016/s0304-3959(99)00242-0 10781906

[42] Delbaere K Crombez G Vanderstraeten G Willems T and Cambier D . Fear-related avoidance of activities, falls and physical frailty. A prospective community-based cohort study. Age Ageing. (2004) 33:368–73. doi: 10.1093/ageing/afh106 15047574

[43] Hadjistavropoulos T Delbaere K Fitzgerald TD . Reconceptualizing the role of fear of falling and balance confidence in fall risk. J Aging Health. (2011) 23:3–23. doi: 10.1177/0898264310378039 20852012

[44] Fried LP Tangen CM Walston J Newman AB Hirsch C Gottdiener J . Frailty in older adults: evidence for a phenotype. J Gerontol A Biol Sci Med Sci. (2001) 56:M146–56. doi: 10.1093/gerona/56.3.m146 11253156

[45] Dent E Martin FC Bergman H Woo J Romero-Ortuno R and Walston JD . Management of frailty: opportunities, challenges, and future directions. Lancet. (2019) 394:1376–86. doi: 10.1016/s0140-6736(19)31785-4 31609229

[46] Yeung SSY Reijnierse EM Pham VK Trappenburg MC Lim WK Meskers CGM . Sarcopenia and its association with falls and fractures in older adults: a systematic review and meta-analysis. J Cachexia Sarcopenia Muscle. (2019) 10:485–500. doi: 10.1002/jcsm.12411 30993881 PMC6596401

[47] Lennon H Kelly S Sperrin M Buchan I Cross AJ Leitzmann M . Framework to construct and interpret latent class trajectory modelling. BMJ Open. (2018) 8:e020683. doi: 10.1136/bmjopen-2017-020683 29982203 PMC6042544

[48] Dombrowsky T . Trajectories of functional decline in older adults: a latent class growth curve analysis. West J Nurs Res. (2023) 45:715–25. doi: 10.1177/01939459231180365 37288523 PMC10359954

[49] Chang H Wang X Wang Z . Latent profile analysis of successful aging among empty nesters in Guiyang, China. Aging Ment Health. (2024) 28:667–74. doi: 10.1080/13607863.2023.2265851 37822072

[50] Scheffer AC Schuurmans MJ van Dijk N van der Hooft T and de Rooij SE . Fear of falling: measurement strategy, prevalence, risk factors and consequences among older persons. Age Ageing. (2008) 37:19–24. doi: 10.1093/ageing/afm169 18194967

[51] Cui Q Zhong Y Gui Y Ma S and Ge Y . Experiences and perceptions of fear of falling in older adults: A systematic review and meta-synthesis of qualitative studies. Geriatr Nurs. (2025) 61:324–35. doi: 10.1016/j.gerinurse.2024.11.003 39579450

[52] Merchant RA Chen MZ Wong BLL Ng SE Shirooka H Lim JY . Relationship between fear of falling, fear-related activity restriction, frailty, and sarcopenia. J Am Geriatr Soc. (2020) 68:2602–8. doi: 10.1111/jgs.16719 32804411

[53] Kim U Lim J Park Y and Bae Y . Predicting fall risk through step width variability at increased gait speed in community dwelling older adults. Sci Rep. (2025) 15:16915. doi: 10.1038/s41598-025-02128-2 40374865 PMC12081882

[54] Ambrose AF Paul G Hausdorff JM . Risk factors for falls among older adults: a review of the literature. Maturitas. (2013) 75:51–61. doi: 10.1016/j.maturitas.2013.02.009 23523272

[55] Cummings SR Nevitt MC Browner WS Stone K Fox KM Ensrud KE . Risk factors for hip fracture in white women. Study of Osteoporotic Fractures Research Group. N Engl J Med. (1995) 332:767–73. doi: 10.1056/nejm199503233321202 7862179

[56] Chua CHM Jiang Y Lim S Wu VX and Wang W . Effectiveness of cognitive behaviour therapy-based multicomponent interventions on fear of falling among community-dwelling older adults: A systematic review and meta-analysis. J Adv Nurs. (2019) 75:3299–315. doi: 10.1111/jan.14150 31287182

[57] Sherrington C Michaleff ZA Fairhall N Paul SS Tiedemann A Whitney J . Exercise to prevent falls in older adults: an updated systematic review and meta-analysis. Br J Sports Med. (2017) 51:1750–8. doi: 10.1136/bjsports-2016-096547 27707740

